# Real-Life Clinical Use and Outcomes of Fusion Imaging-Guided Percutaneous Microwave Ablation of Hepatocellular Carcinoma: Experience from Two Italian Centers

**DOI:** 10.3390/diagnostics15131573

**Published:** 2025-06-20

**Authors:** Pierpaolo Biondetti, Francesco Cicchetti, Gaetano Valerio Davide Amato, Velio Ascenti, Niccolò Finardi, Jacopo Tintori, Francesco Ugo Iovino, Carolina Lanza, Salvatore Alessio Angileri, Pierluca Torcia, Anna Maria Ierardi, Giacomo Vignati, Lorenzo Giovanni Monfardini, Gianpaolo Carrafiello

**Affiliations:** 1 Department of Diagnostic and Interventional Radiology, Foundation IRCCS Cà Granda—Ospedale Maggiore Policlinico, Via Francesco Sforza 35, 20122 Milan, Italy; pierpaolo.biondetti@policlinico.mi.it (P.B.); velio.ascenti@unimi.it (V.A.); francescoiovino.st@gmail.com (F.U.I.); carolina.lanza@unimi.it (C.L.); alessioangileri@gmail.com (S.A.A.); pierluca.torcia@policlinico.mi.it (P.T.); annamaria.ierardi@policlinico.mi.it (A.M.I.); gianpaolo.carrafiello@unimi.it (G.C.); 2 Postgraduate School in Radiodiagnostics, Università degli Studi di Milano, 20122 Milan, Italy; francesco.cicchetti@unimi.it (F.C.); niccolo.finardi@unimi.it (N.F.); jacopo.tintori@unimi.it (J.T.); giacomo.vignati@unimi.it (G.V.); 3 Divisione di Radiologia Ospedale San Giuseppe, IRCCS Multimedica, Via San Vittore, 12, 20123 Milan, Italy; lorenzo.monfardini@multimedica.it; 4 Department of Oncology and Hemayo-Oncology, Università degli Studi di Milano, 20122 Milan, Italy

**Keywords:** HCC, microwave ablation, ultrasound fusion imaging, imaging-guided ablation, liver disease

## Abstract

**Background:** Hepatocellular carcinoma (HCC) is a major cause of cancer-related death worldwide. Percutaneous thermal ablation is an effective treatment, but standard ultrasound (US) guidance is limited in cases of inconspicuous nodules. Ultrasound fusion imaging (USFI), which overlays cross-sectional imaging onto real-time US is an emerging technique that improves tumor visibility and technical feasibility. This study reports real-life outcomes of USFI-guided microwave ablation (MWA) for HCC in two Italian centers. **Materials and Methods:** In this multicentric retrospective study, 56 patients with 73 poorly or non-visible HCC nodules underwent USFI-guided percutaneous MWA with no visibility or poor visibility on B-mode US. Technical success, complications, and local tumor control were evaluated, with follow-up imaging at 1 month and every 3 months thereafter. **Results:** Complete response (CR) at 1 month was observed in 78.1% of nodules, with residual disease (RD) more common in poorly visible nodules than non-visible nodules (18.1% vs. 4.2%, *p* = 0.019). During a median 13-month follow-up, local tumor progression (LTP) occurred in 9.6% of patients. No significant association was found with difficult tumor location. **Conclusions:** USFI-guided MWA is a safe and effective option for treating HCC nodules not adequately visualized with conventional US, expanding eligibility to complex cases.

## 1. Introduction

Hepatocellular carcinoma (HCC) is the third leading cause of cancer-related mortality globally [1]. Thermal ablation is currently an established treatment modality for patients with HCC [2]. In recent years, the detection of tumors small enough to be treated with thermal ablation has increased [3,4]; conversely, the outcomes of thermal ablation for liver tumors have improved, particularly when performed percutaneously [5]. Imaging guidance serves as the cornerstone of every percutaneous intervention, which has also benefitted from significant technological advancements. Ultrasound (US) is widely regarded as the preferred imaging modality for the ablation of hepatic tumors due to its broad availability and the critical advantage of real-time imaging [6]. However, US can be limited by deep lesions, large patients, or poor tumoral sonographic conspicuity. Furthermore, the livers of patients with HCC are often affected by chronic changes, resulting in diffuse parenchymal inhomogeneity and pseudo-lesion formation [7].

To overcome these challenges, interventional radiologists can take advantage of advanced US guidance modalities, including contrast-enhanced ultrasound (CEUS) and ultrasound fusion imaging (USFI). Fusion imaging involves registering two or more imaging datasets from different modalities, whether obtained simultaneously or at different times. When applied to US in interventional radiology, it typically refers to the overlay of pre-procedural cross-sectional studies onto real-time ultrasound images, achievable manually or automatically using anatomical landmarks, pathological landmarks, or sensor coils [5]. USFI is performed to combine the clear visualization of anatomy and targets offered by cross-sectional imaging with the benefits of real-time ultrasound guidance.

The existing literature, coming mainly from the Eastern world, has demonstrated the utility of USFI in treating patients with HCC [8,9,10]. USFI appears to enhance the operator’s confidence in planning and performing procedures, thus facilitating the treatment of invisible or poorly detectable lesions on simple B-mode US [11].

Despite the existing evidence, this technique is still not widely adopted, and reports from the Western world are still a minority.

In this study we aim to report our real-life experience with the use of USFI for percutaneous thermal ablation of HCC tumors poorly or non-identifiable with simple US.

## 2. Materials and Methods

### 2.1. Study Design and Population Characteristics

This multi-centric retrospective study, in accordance with the ethical standards of the institutional research committee and the 1964 Helsinki declaration, enrolled patients with HCC treated with percutaneous MWA guided by USFI at Fondazione IRCCS Cà Granda—Ospedale Maggiore Policlinico (*n* = 42) and Ospedale MultiMedica San Giuseppe (*n* = 14), both located in Milan, from January 2021 to December 2024.

All patients included in the study met the following inclusion criteria:

- Age ≥ 18 years.
- Pathologic or typical imaging-based diagnosis of HCC.
- Solitary HCC measuring ≤ 3.5 cm or ≤3 HCC lesions each measuring ≤ 3.0 cm.
- Availability of follow-up imaging ≥ 1-month post-ablation.
- Availability of pre-procedural imaging performed within 1 month before procedure.
- ≥1 poorly visible or non-visible HCC nodule with B-mode US that was treated with USFI.

HCC diagnosis was based on typical CT/MR imaging features or pathologic confirmation according to EASL guidelines [12].

Patients in whom the microwave antenna was positioned or corrected with guidance modalities other than USFI were excluded. In cases of multifocal disease, the presence of a single poorly visible or non-visible HCC nodule resulted in the treatment of all remaining nodules with USFI-guided MWA.

Eligibility for tumor ablation was determined, following major societies’ guidelines [12,13], based on standard criteria such as disease stage, comorbidity, patient age, and refusal of surgical intervention.

A multidisciplinary team involving hepatologists, surgeons, interventional radiologists, and radiation oncologists indicated treatment for each patient.

In our institutions, each patient eligible for percutaneous liver thermal ablation undergoes a pre-procedural outpatient US examination to establish whether the target lesion is visible, poorly visible, or non-visible under simple B-mode. Nodules are classified as poorly visible when they are partially visible even during deep inspiration, or if they exhibit poor conspicuity or have indistinct tumor margins. If no focal change in the sonographic properties of the liver is detected, tumors are classified as non-visible [14].

Electronic medical records were reviewed to collect epidemiological and patient-related data, including sex, age at the time of treatment, presence of cirrhosis, etiology of liver disease, Child–Pugh score, BCLC stage, and previous hepatic treatments.

Pre-procedural CECT and CEMR were evaluated to assess tumor-related data, including the number of nodules, maximum axial dimension, hepatic segmental location, and challenging localization.

Nodules were classified as challenging when located near potentially delicate structures, specifically within 5 mm of the heart, diaphragm, gallbladder, main bile duct, vessels > 3 mm in diameter, or the hepatic capsule.

In patients with multifocal tumors all nodules were treated in the same session.

### 2.2. Procedure

Risks and benefits of the proposed treatment were discussed with each patient before the procedure, and informed consent was obtained.

Coagulation tests were within the normal range for all patients; patients undergoing anticoagulant and/or antiplatelet therapy were managed as specified by interventional society documents [15].

Each patient received antibiotic prophylaxis.

All procedures were performed in an angiographic suite with anesthesiologic support and with continuous monitoring of vital parameters by one of six interventional radiologists that had no experience with USFI at the beginning the study period.

Before the procedure, pre-procedural CECT or CEMR images were uploaded into the US machine (Epiq 5, Philips Medical Systems, Best, The Netherlands).

All patients were positioned supine and kept in light sedation for the first part of the procedure to allow patient cooperation, including breath holding which is required for the fusion process.

**Figure 1:**
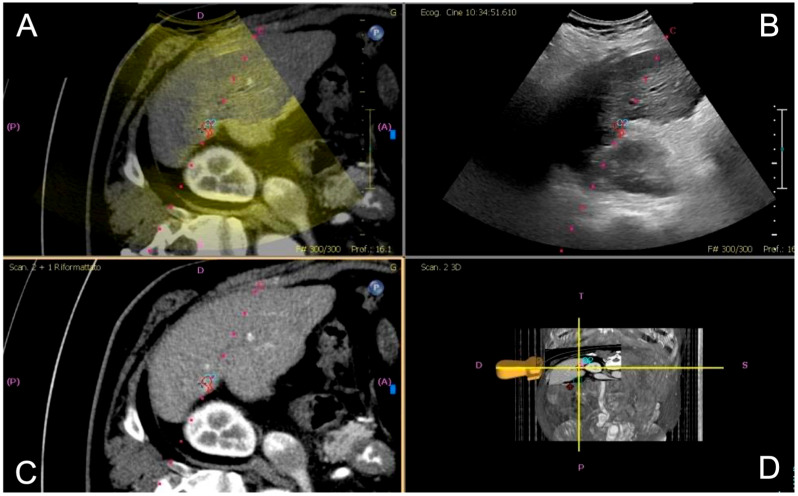
Image fusion workflow for percutaneous ablation planning. After co-registration of pre-procedural CT images with real-time ultrasound (US), the integrated display includes: fused CT–US images with the US superimposed on the CT images (**A**), real-time US alone (**B**), CT images alone (**C**), and 3D position of the US probe (**D**). The target lesion is highlighted with a pink circle, aiding in precise localization for intervention.

Fusion imaging was performed with electromagnetic tracking using automatic vessel registration via dedicated software (PercuNav System, Software vers. 7.0.8, Philips Medical Systems, Netherlands).The process involves acquiring an ultrasound scan of the liver to create a 3D dataset registered by a software to cross-sectional images based on the hepatic vessels (Figure 1). If automatic registration was not judged adequate, some manual adjustments were performed.

**Figure 2:**
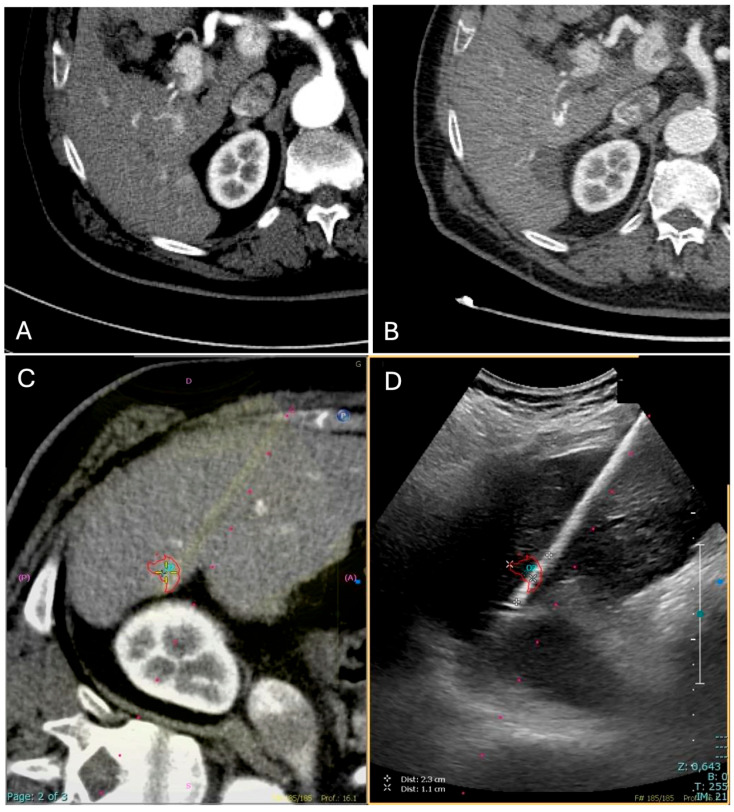
Contrast-enhanced CT (CECT) in the arterial phase demonstrates a newly diagnosed HCC located in segment VI of the liver, near the hepatic capsule. (**A**) The lesion shows intense arterial-phase hyperenhancement (wash-in), a typical imaging hallmark of HCC. (**B**) One-month follow-up CECT shows no residual enhancement, consistent with complete response to treatment. (**C**) CT–US fusion image following co-registration, enabling precise visualization of the lesion (highlighted by a red target). (**D**) The corresponding real-time US image during the ablation procedure, with the lesion and ablation needle clearly visualized (highlighted by a red target).

Once the microwave 13.5 G antenna (Emprint Microwave Ablation System, Medtronic, and Covidien, Boulder, CO, USA) was in place, sedation was deepened and ablation was performed with a power of either 100 or 150 W for a time established by the operator based on the tumor size and the microwave manufacturer data; all cases were ended with track ablation (Figure 2).

If the clinical course was uncomplicated, patients were hospitalized for one night after the procedure and discharged the following day.

Technical success was defined as performing the registration process correctly and the entire procedure under USFI guidance.

Any complication during or after procedure was registered [16].

### 2.3. Follow-Up and Outcome

Each patient underwent radiological follow-up contrast-enhanced CT after 1 month and every 3–4 months thereafter.

The primary endpoint of this study was to evaluate the outcome of the procedure through local tumor control, measured as residual disease (RD) or local tumor progression (LTP).

Complete response (CR) was defined as imaging evidence of complete tumor ablation. Residual disease (RD) referred to the presence of viable tumor tissue at the ablative margin on the first 1-month follow-up imaging. Local tumor progression (LTP) was defined as the reappearance of tumor after at least one contrast-enhanced follow-up scan had shown no residual viable tumor at the ablative margin [17,18]. Therefore, LTP was assessed only in patients who did not present with RD at the 1-month follow-up. Follow-up time was defined as the time interval between the treatment date and the most recent imaging examination at our institution or institutional visit in which imaging from elsewhere was assessed.

In the case of tumor detection during follow-up, the indication for a new treatment was discussed in a multidisciplinary setting.

Patients were censored in case of liver transplant, systemic therapy, or death [19].

### 2.4. Statistical Analysis

Quantitative variables were reported as mean and median values, with corresponding minimum and maximum values, while categorical variables were presented as absolute counts and percentages.

To assess the statistical association between nodule visibility and response at the 1-month follow-up, the single visible nodule present in the dataset was excluded from the analysis.

Pearson chi-square test was used to evaluate the association between local response at 1 month and nodule visibility, as well as the association between local response at 1 month and difficult site location of the nodule.

The same statistical approach was applied to assess the associations between the total follow-up local response and nodule US visibility, and between total follow-up local response and difficult tumor site, after excluding those patients that had residual disease at the first follow-up examination.

Binary logistic regression was conducted to investigate factors associated with complete radiological response (CR) compared to residual disease (RD). Model adequacy was assessed using the likelihood-ratio test, pseudo-R^2^ statistics, and the Hosmer–Lemeshow goodness-of-fit test. Statistical analyses were performed using IBM SPSS software (version 29.0.2.0, IBM Corp., Armonk, NY, USA).

## 3. Results

### 3.1. Population

**Figure 3:**
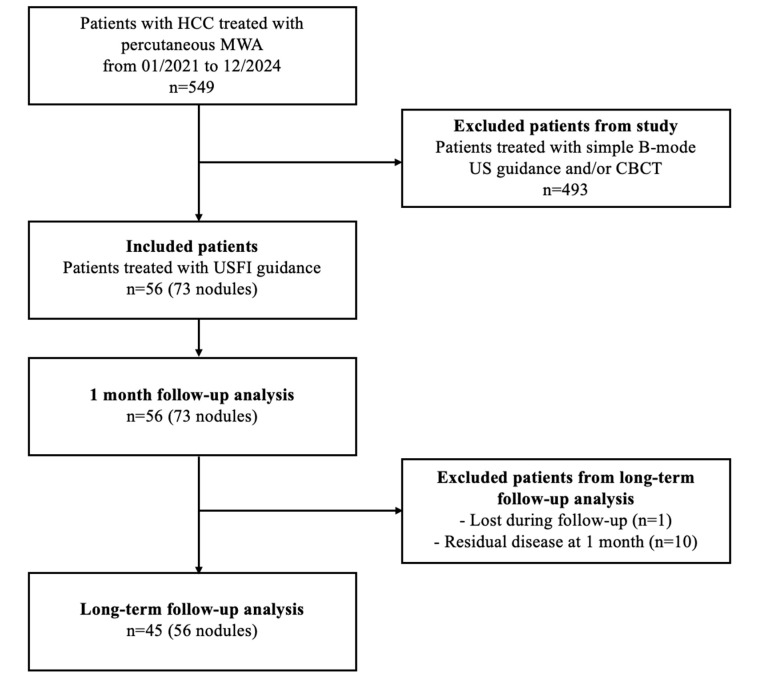
Flow diagram of patient selection and inclusion in the study cohort.

Between January 2021 and December 2024, a total of 549 patients underwent percutaneous MWA for HCC nodules in the two institutions. Ultrasound fusion imaging (USFI) was used in 56/549 patients (9.8%), 42 from Fondazione IRCCS Cà Granda—Ospedale Maggiore Policlinico and 14 from Ospedale MultiMedica San Giuseppe, with a total of 73 nodules; this population was enrolled in our study (Figure 3).

The mean age of included patients was 70.5 years (range 39–91 years). Most patients (96.4%) had liver cirrhosis with the most common etiology of liver disease being HCV, accounting for 51.8% of cases, followed by alcoholic cirrhosis (19.4%) and HBV or HBV/HDV coinfection (19.4%). Most patients were classified as Child–Turcotte–Pugh A (83.9%) and BCLC A (78.6%). The other causes of cirrhosis, along with the Child–Turcotte–Pugh classification and the BCLC groups, are listed in Table 1.

Tumor-related data are summarized in Table 2.

Most patients (75%) had monofocal disease, whereas a minority had one (19.6%) or three (5.4%) HCC nodules. The mean diameter of tumors was 15.5 mm (range 7–33 mm). The VIII segment was the most frequently involved (42.5%).

Among target nodules, 37% had already been subject to treatment attempts and therefore were residual disease. HCC nodules were visible or poorly visible in 43.8% and 54.8% of cases, respectively. A single nodule that was visible on B-mode US was treated with USFI in one patient that had concurrent non-visible tumors.

More than half of the nodules (53.4%) were in challenging areas, particularly near the liver capsule (31.5% of cases). A thermal ablation power of 100 W was applied to 74% of the nodules, while 26% were treated with a thermal ablation power of 150 W.

### 3.2. Complications and Follow-Up

The follow-up period varied from 1 to 40 months, with a mean of 14.2 months and a median of 13 months.

There were no ablation-related deaths. Most patients (87.5%) did not experience any complications and only one patient experienced major complications. The most common complication following ablation was pain, which affected four patients. Fever and post-ablation syndrome were registered in two cases. There was only one case which involved a hemorrhagic complication characterized by hemoperitoneum without evidence of active arterial bleeding, which was monitored in the following days and resolved spontaneously without any intervention.

At 1-month follow-up we registered a complete response in 78.1% of cases, while residual disease was observed in 21.9%.

During the entire follow-up time, complete local response was registered in 67.1% and LTP in 9.6% of patients. Mean time to local tumor progression was 12.1 months (range 3–33 months). (Table 3)

As shown in Table 4, at the 1-month follow-up, poorly visible nodules demonstrated a higher residual disease rate compared with non-visible nodules (18.1% vs. 4.2%; *p* = 0.019). However, over the total follow-up period, the rate of local tumor progression (LTP) was lower for poorly visible nodules than for non-visible ones (1.4% vs. 8.3%; *p* = 0.010). Notably, while nodule visibility influenced treatment outcomes, tumor location was not associated with local treatment response either at the 1-month evaluation or during the total follow-up period (Table 5).

After exclusion of the fully visible nodule, 72 lesions were evaluable. The multivariable model approached overall significance (*p* = 0.061) and demonstrated acceptable calibration (*p* = 0.304). The only independent predictor was partial ultrasound visibility, which correlated with an 84% decrease in the chances of a complete response (OR = 0.16, 95% CI 0.04–0.69, *p* = 0.014). In the adjusted analysis, lesion size, challenging location, and previous liver treatment were not statistically significant (Table 6).

## 4. Discussion

Imaging guidance represents a key to success in interventional radiology. Percutaneous ablation of HCC is now a standard procedure performed in many cases under B-mode guidance, but this imaging modality has inherent limitations in its ability to correctly visualize some of the target nodules.

To overcome this issue, advanced ultrasound guidance modalities, including USFI, are available today [20,21].

In this study we reported the real-life clinical use, safety, and efficacy of percutaneous thermal ablation of liver HCC from two different Italian centers using USFI as imaging guidance.

This technique is used for a minority of patients with HCC in which indication is given to treatment with percutaneous image-guided thermal ablation; this selected population, composed in our practice of patients with tumors either poorly visible or non-visible with standard US, represented less than 10% of the patients treated (9.8%).

Of note, tumors treated with percutaneous thermal ablation under USFI received previous treatments in a high proportion of cases (37%); this might be expected as an altered anatomy from prior treatments makes nodule identification more difficult, or impossible in some cases, with standard US, and at the same time highlights the difficulty of treating these lesions.

Interestingly, in our population, tumors in the eighth segment were the most frequently treated using USFI, likely due to its relative sonographic inaccessibility caused by distance from the ultrasound probe and limited acoustic windows.

Another important point is that a high proportion of tumors treated with USFI were not visible at all with standard US (43%); these tumors, excluding MR guidance which is expensive, need specialized equipment and personnel and therefore USFI guidance is of very limited actual use across the world. However, it is very important to offer curative treatments such as ablation instead of leaving only the option to convert to TACE.

Keeping this in mind, we observed a complete local response to treatment at 1 month in 78.1% of tumors, which is slightly lower than that described in a recent systematic review by Calandri et al. that reported 1-month complete local response rates of between 84.3% and 100% [22]. This may be attributed to several technical and clinical factors. Firstly, in our institution, ablations are performed under light/deep sedation to extend the indication to patients who cannot tolerate general anesthesia, a factor that makes the registration process more challenging and leads to a variability in the registration of images during the breathing phase. Secondly, the populations examined across studies were different: in our study, as mentioned above, there was a high rate (43%) of nodules classified as not visible, 37% of patients had previously undergone locoregional treatments, and a high number of patients had an advanced stage of chronic liver disease. All these factors reflect the challenging population treated with USFI in our referral center. Additionally, unlike most studies where patients underwent supplemental ablation in cases of residual disease, therefore resulting in secondary efficacy, in this study we considered the efficacy of only the first ablation treatment session (primary efficacy).

Interestingly, we registered a high 1-month complete local response rate (95.8%) when considering only the subgroup of non-visible target tumors by simple B-mode US. This is similar to what was reported by Ahn et al., who, among 216 patients with 245 HCCs, described a 1-month complete local response rate of 96.1% for non-visible tumors, which was a similar result similar to the 97.6% complete local response rate reported for visible nodules [14].

Regarding LTP rate during the entire follow-up time, our registered rate was 9.6% which is consistent with data reported in literature [22].

Regarding the association between local treatment response at 1 month and nodule visibility, the fact that poorly visible nodules were associated with a higher rate of residual disease and lower odds of complete ablation when compared to totally non-visible nodules (18.1% vs. 4.2%, respectively, *p* = 0.019) was unexpected, but the reason could be that since the treatment of non-visible nodules relied totally on USFI, in this group, the operators probably chased the highest possible degree of accuracy in the coregistration of CT/MR and US images; in contrast, the “poorly visible” group probably suffered from operator bias and overconfidence in the US B-mode, resulting in less accuracy during the registration process and therefore worse outcomes [14,23]. However, the statistical significance of these data was certainly limited by the low number of patients.

When interpreting the difference observed for LTP rates based on nodule visibility, it must be kept in mind that the analysis was made only in patients that had an initial complete response, for both poorly visible and non-visible nodule groups, and this further limited the significance of results as numbers were even smaller. Nevertheless, in this case a higher rate of LTP during follow-up for non-visible tumors when compared to poorly visible ones (8.3% vs. 1.4%, *p* = 0.010) was not unexpected.

There was no difference in this study regarding ablation outcomes between tumors in difficult and non-difficult locations, both in terms of residual disease at 1 month (12.3% vs. 9.6%, *p* = 0.798) and LTP during follow-up (5.5% vs. 4.1%, *p* = 0.839), suggesting that anatomical challenges did not compromise the efficacy of treatment and that USFI may be applied to all cases.

Lastly, in our experience, percutaneous MWA ablation of HCC guided by USFI was safe and well tolerated, as demonstrated by the vast majority (87.5%) of cases showing no complications, with only one major complication and no deaths.

This study had several limitations, including the limited number of patients, the short follow-up period, the absence of a comparator group, its retrospective nature, and the absence of data regarding the quality of the coregistration of CT/MR images to US images for each patient.

## 5. Conclusions

In conclusion, in real-life clinical practice, USFI-guided percutaneous thermal ablation of liver HCCs is a safe and effective treatment. USFI serves as an essential tool in complex cases, particularly for nodules that are non-visible on conventional ultrasound or located in challenging anatomical positions. Its use enables the successful ablation of tumors that might otherwise be considered inaccessible, potentially avoiding the need for less effective endovascular treatments. Prospective, possibly randomized, trials are needed to better understand which patients would benefit most from these technologies.

## Figures and Tables

**Table 1:** Patient characteristics.

Characteristic	Value (*n* = 56 Patients)
Age	Mean/median 70.46/69.5 (39–91)
Sex	
Male	44 (78.6%)
Female	12 (21.4%)
Cirrhosis	
Yes	54 (96.4%)
No	2 (3.6%)
Etiology	
HCV	29 (51.8%)
NAFLD	17 (30.4%)
Alcohol	11 (19.6%)
HBV or HBV/HDV	11 (19.6%)
Child–Pugh	
A	47 (83.9%)
B	6 (10.7%)
C	1 (1.8%)
Not classified	2 (3.6%)
BCLC grade	
0	5 (8.9%)
A	44 (78.6%)
B	6 (10.7%)
C	1 (1.8%)

Abbreviations: HBV, hepatitis B virus; HDV, hepatitis D virus; HCV, hepatitis C virus; NAFLD, non-alcoholic fatty liver disease.

**Table 2:** HCC nodule characteristics among patients included in the study.

Characteristic	Value (*n* = 73 Nodules)
Number of HCC nodules	
1	42 (75%)
2	11 (19.6%)
3	3 (5.4%)
Previous treatment	
Yes	27 (37%)
No	46 (63%)
Nodule dimension (mm)	Mean/median 15.5/15 (5–33)
Nodule dimension (n)	
<10 mm	6 (8.2%)
10–20 mm	50 (68.5%)
>20 mm	17 (23.3%)
Hepatic segments	
VIII	31 (42.5%)
VI	13 (17.8%)
IV	9 (12.3%)
V	6 (8.2%)
VII	6 (8.2%)
I	5 (6.8%)
III	3 (4.1%)
Difficult location	
Yes/no	39 (53.4%)/34 (46.6%)
Subcapsular	23 (31.5%)
Diaphragm	6 (8.2%)
Diaphragm and subcapsular	4 (5.5%)
Vessel and subcapsular	4 (5.5%)
Vessel	2 (2.7%)
US visibility	
Not visible	32 (43.8%)
Poorly visible	40 (54.8%)
Visible	1 (1.4%)

**Table 3:** Outcomes.

Outcome Response	Value (*n* = 73 Nodules)
Local Ablation Response (1 month)	
CR	57 (78.1%)
RD	16 (21.9%)
Local Ablation Response	
CR	49 (67.1%)
LTP	7 (9.6%)
RD and Censored Cases	17 (23.3%)
Time of LTP (months)	Mean/median 12.1/9 (3–33)

Abbreviations: CR, complete response; RD, residual disease; LTP, local tumor progression.

**Table 4:** Correlation between US visibility and local response at 1 month and at different times of follow-up evaluation using the Pearson chi-square test.

	Non-Visible Nodules	Poorly Visible	*p*-Value
Local Ablation Response (1 month)			
RD	3 (4.2%)	13 (18.1%)	0.019
CR	29 (40.3%)	27 (37.5%)	
Local Ablation Response			
LTP	6 (8.3%)	1 (1.4%)	0.010
CR	23 (31.9%)	26 (36.1%)	

**Table 5:** Correlation between nodule location and local response at 1 month and at different times of follow-up evaluation using the Pearson chi-square test.

	Difficult Location	Not Difficult	*p*-Value
Local Ablation Response (1 month)			
RD	9 (12.3%)	7 (9.6%)	0.798
CR	30 (41.1%)	27 (37.0%)	
Local Ablation Response			
LTP	4 (5.5%)	3 (4.1%)	0.839
CR	25 (34.2%)	24 (32.9%)	

**Table 6:** Independent predictors of complete radiological response (CR) after percutaneous ablation (*n* = 72).

Predictors	OR Adjusted (95% CI)	*p*-Value
Previous liver treatment	0.81 (0.22–3.06)	0.758
Lesion size, cm (per 1 cm increase)	0.92 (0.82–1.03)	0.153
Difficult location	0.61 (0.18–2.10)	0.434
Poor ultrasound visibility	0.16 (0.04–0.69)	0.014

## Data Availability

The raw data supporting the conclusions of this article will be made available by the authors on request.

## Abbreviations

The following abbreviations are used in this manuscript:

CECT	Contrast-enhanced computed tomography
CEMR	Contrast-enhanced magnetic resonance
CR	Complete response
CT	Computed tomography
HBV	Hepatitis B virus
HCC	Hepatocellular carcinoma
HCV	Hepatitis C virus
HDV	Hepatitis D virus
NAFLD	Non-alcoholic fatty liver disease
LTP	Local tumor progression
MR	Magnetic resonance
MWA	Microwave ablation
RD	Residual disease
TACE	Transarterial chemoembolization
US	Ultrasound
USFI	Ultrasound fusion imaging
